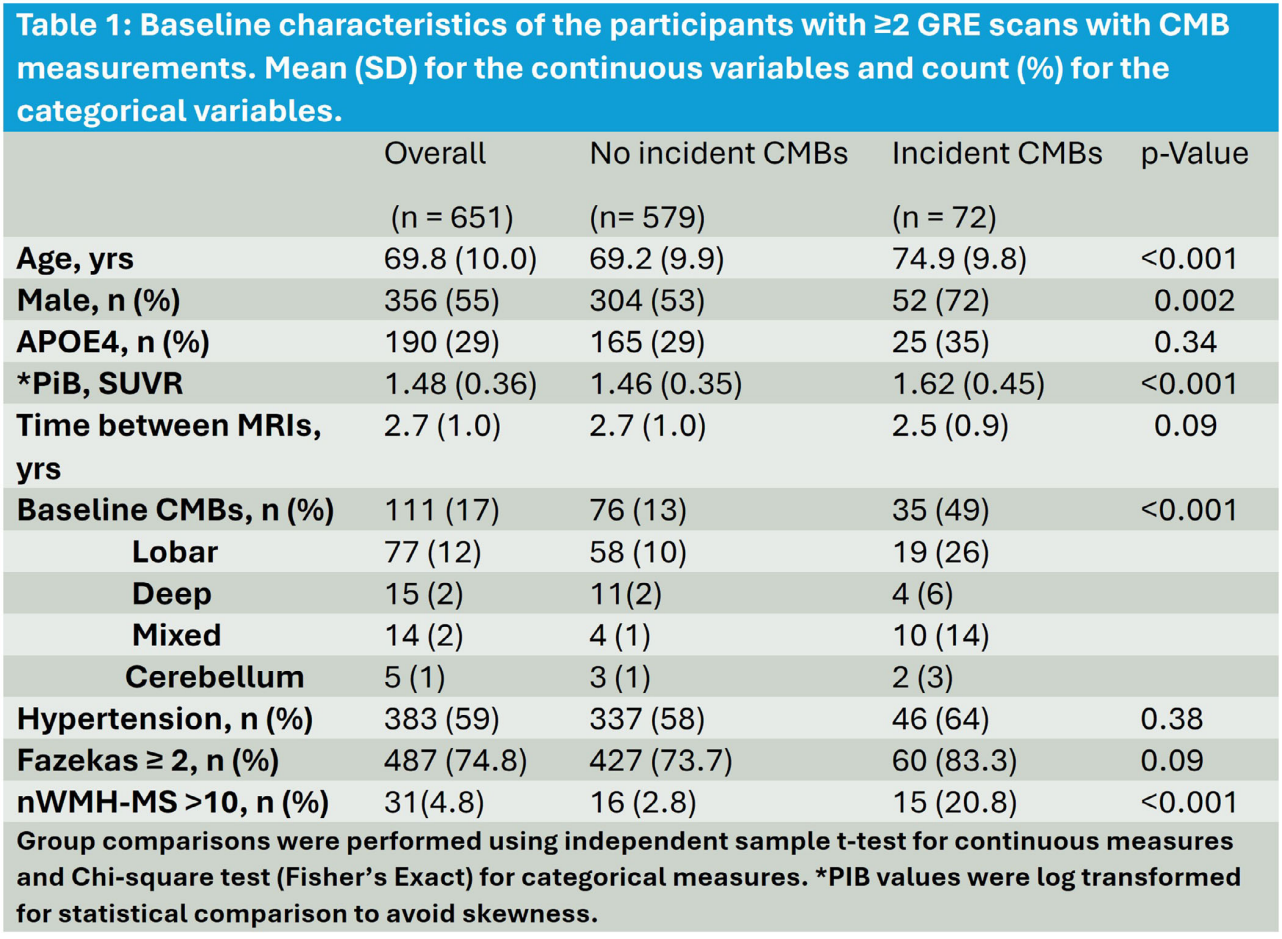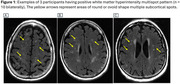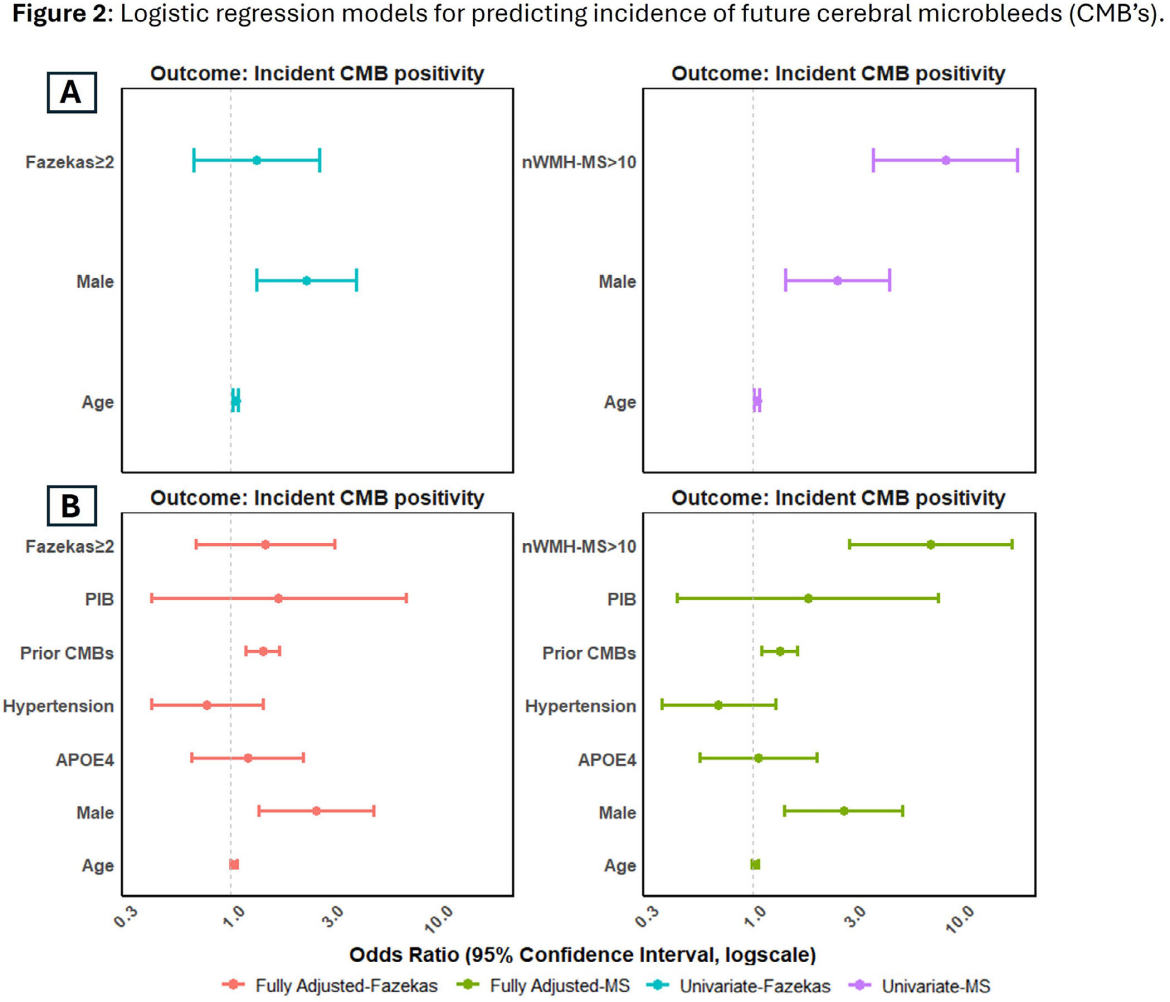# Multispot white matter hyperintensity pattern as a surrogate marker of cerebral amyloid angiopathy in the population

**DOI:** 10.1002/alz70856_103092

**Published:** 2025-12-26

**Authors:** Sheelakumari Raghavan, Jonathan Graff‐Radford, Jeffrey L. Gunter, Scott A. Przybelski, Anthony J. Spychalla, Petrice M Cogswell, Vijay K. Ramanan, Ronald Petersen, Clifford R. Jack, Prashanthi Vemuri

**Affiliations:** ^1^ Mayo Clinic, Rochester, MN, USA; ^2^ Department of Neurology, Mayo Clinic, Rochester, MN, USA; ^3^ Department of Radiology, Mayo Clinic, Rochester, MN, USA; ^4^ Department of Quantitative Health Sciences, Mayo Clinic, Rochester, MN, USA; ^5^ Mayo Clinic Alzheimer's Disease Research Center, Rochester, MN, USA; ^6^ Mayo Clinic, Radiology, Rochester, MN, USA

## Abstract

**Background:**

White matter hyperintensity topography, particularly posterior confluent and subcortical multispot (WMH‐MS), has been implicated as surrogate imaging biomarker of cerebral amyloid angiopathy (CAA). Usefulness of WMH‐MS for prediction of future cerebral microbleeds (CMB's) has significant clinical implications for anti‐amyloid therapy risk assessment. Our primary goal was to identify the prevalence of positive WMH‐MS status and their utility for predicting the incident CMBs in a population‐based sample. Our secondary goal was to evaluate its usefulness after accounting for amyloid (surrogate for CAA) and WMH burden (surrogate of small vessel disease) which are both risk factors for CMBs.

**Method:**

We identified 651 participants (mean age (SD) 69.8 (10.0) years, 55% male) from the Mayo Clinic Study of Aging, who had 3T baseline whole brain fluid‐attenuated inversion recovery (FLAIR) MRI and at least two T2*‐weighted gradient recalled echo (GRE) sequences (concomitant with or after FLAIR) which were used to ascertain CMBs. Amyloid positron emission tomography (PiB‐PET) scans were available in 87% of the sample. Visual reads on FLAIR scans were performed using a 4‐point Fazekas rating scale of periventricular and deep WMH (positivity: Fazekas ≥ 2) and a 3‐point MS severity visual scale (positivity: nWMH‐MS > 10 circular or ovoid WMH spots, Figure 1) according to established criteria. Using univariate and multivariable logistic regression, we assessed the independent association of WMH‐MS > 10 and Fazekas ≥ 2 for prediction of incident CMB's.

**Result:**

The frequency of WMH‐MS pattern was greater in those who developed an incident CMB compared to those who did not develop a CMB (20.8% vs. 2.8%, *p* < 0.001). Older age and male sex were predictive of incident CMBs (Figure 2A). In univariate models, presence of WMH‐MS pattern but not Fazekas was associated with incident CMBs after adjusting for age and sex. The associations remained significant after additional adjustment for *APOE4*, hypertension, number of baseline CMBs (Prior CMB), and amyloid effects (Figure 2B).

**Conclusion:**

Visually identified WMH‐MS patterns on FLAIR‐MRI were predictive of incident CMBs in this population and may be useful markers of CAA. Future studies evaluating its utility as an additional biomarker of amyloid related imaging abnormalities in clinical trials are necessary.